# Reference genes for accessing differential expression among developmental stages and analysis of differential expression of OBP genes in *Anastrepha obliqua*

**DOI:** 10.1038/srep17480

**Published:** 2016-01-28

**Authors:** Aline Minali Nakamura, Samira Chahad-Ehlers, André Luís A. Lima, Cristiane Hayumi Taniguti, Iderval Sobrinho Jr., Felipe Rafael Torres, Reinaldo Alves de Brito

**Affiliations:** 1 Departamento de Genética e Evolução, Universidade Federal de São Carlos, São Carlos, Brazil; 2 Universidade Federal de Goiás, Jataí, Brazil

## Abstract

The West Indian fruit fly, *Anastrepha obliqua*, is an important agricultural pest in the New World. The use of pesticide-free methods to control invasive species such as this reinforces the search for genes potentially useful in their genetic control. Therefore, the study of chemosensory proteins involved with a range of responses to the chemical environment will help not only on the understanding of the species biology but may also help the development of environmentally friendly pest control strategies. Here we analyzed the expression patterns of three OBP genes, *Obp19d_2*, *Obp56a* and *Obp99c*, across different phases of *A. obliqua* development by *q*PCR. In order to do so, we tested eight and identified three reference genes for data normalization, *rpl17*, *rpl18* and *ef1a*, which displayed stability for the conditions here tested. All OBPs showed differential expression on adults and some differential expression among adult stages. *Obp99c* had an almost exclusive expression in males and *Obp56a* showed high expression in virgin females. Thereby, our results provide relevant data not only for other gene expression studies in this species, as well as for the search of candidate genes that may help in the development of new pest control strategies.

The genus *Anastrepha* has over 235 species^1^ most of them endemic to the Neotropics. Only a few of these species, mostly in the *fraterculus* group, are agricultural pests, including the West Indies fruit fly *Anastrepha obliqua* (Diptera, Tephritidae). Because of the vast damage they inflict to several different fruit crops, they are of great economic importance, which elicit the development of several pest management strategies. Genetic control technique’s, as the Sterile Insect Technique (SIT) using gamma radiation, have been used to control natural populations of other tephritids such as *Ceratitis capitata*^2^. However, radiation leads to side effects^3^,^4^, decreasing the technique efficiency. Thus, the search of candidate genes that may lead to more competitive transgenic organisms may be an alternative for improvement of SIT programs.

A number of candidate genes has already been explored and promising results are reported for some, such as *Astra* and *Astra-2* genes for the Caribbean fruit fly *Anastrepha suspensa*^5^. This encourages the search to identify genes related to reproduction and species recognition in this genus, which may be a priori good candidate genes for genetic control of pest populations. Because the olfactory system participates in important reproductive aspects, such as choosing a partner for mating and searching for oviposition sites^6^,^7^,^8^,^9^,^10^, genes that code for proteins involved in olfactory reception may prove useful in this regard as well.

From chemical stimulus to behavioral response, the olfactory process involves the capture, binding, transport and inactivation of odors; the activation of receptors and signal transduction; and the perception of odor by processing the signals at several levels of the central nervous system^6^,^7^. Thus, chemoreception participates in a range of responses to the chemical environment for animal survival, such as chemical cues for foraging behavior and food selection, predator avoidance, host plant recognition for oviposition and larval feeding, selection of mating partners, maternal behavior, and kin recognition^11^. In insects, the beginning of the transduction cascade of olfactory signals is the solubilization and transport of the chemical signals through the aqueous lymph of insect’s sensilla to the olfactory receptors (ORs). The Odorant Binding Proteins (OBPs) mediate these processes^12^, making the interaction between the environment and the insect, and determining the behavioral response. Hence, OBPs have been recognized as good candidates for genetic control programs of insect pests^13^,^14^ and hematophagous insect vectors^15^,^16^,^17^,^18^.

One important step in the process of prospecting genes for genetic control is the understanding of expression patterns of candidate genes in the target organism. Quantitative polymerase chain reaction (_*q*_PCR) has become an invaluable tool in studies of gene expression due to its sensitivity and precision, enabling the detection of different contrasts among RNA samples. Differential expression of some dipterans’ OBP family members has been studied by _*q*_PCR in different tissues^14^ and in different adult life stages^13^.

In this study, the goal was to assess by _*q*_PCR the expression profile of three OBPs genes, *Obp19d_2*, *Obp56a* and *Obp99c*, at different developmental stages on the West Indies fruit fly. These genes were chosen due to their differential expression between virgin and post-mating males in a previous in silico analysis, suggesting them as potential targets for further studies. However, there is a dearth of information regarding gene expression, as well as good reference genes for data normalization in _*q*_PCR expression studies in most animal species, let alone for *A. obliqua*. Thus, in addition to an analysis of OBPs’ expression, this study also aimed to determine a set of reliable reference genes for expression studies across different developmental stages for the West Indies fruit fly. In order to do so, we investigate patterns of gene expression for eight potential reference genes using _*q*_PCR: *ribosomal protein L18* (*rpl18*), *β-Tubulin* (*btub*), *elongation factor 1α* (*ef1a*), *ribosomal protein S17* (*rps17*), *glyceraldehyde-3-phosphate dehydrogenase* (*gapdh*), *actin* (*act*), *Syntaxin* (*Syx*) and *Troponin C* (*TpnC*) . Our results indicate that the genes *rpl18*, *rps17* and *ef1a* show the highest expression stability throughout development, making them potentially reliable reference genes for gene expression studies in *A. obliqua*. Our results also show that some OBP genes are differentially expressed across different genders and reproductive stages, which make them good candidates for future application in genetic control.

## Results

### Efficiency of candidate reference genes

A quantitative study of relative gene expression requires normalization of data for target genes against a set of reference genes that are used as internal standards and are evenly expressed among tested conditions. Therefore, we investigated gene expression patterns of eight potential reference genes in different *A. obliqua* developmental stages. To determine the best primer concentrations, we tested three different concentrations in several primer combinations. The optimal concentration was 0.3 *μ*M compared to 0.1 *μ*M and 0.15 *μ*M. For sensitivity assays, all genes tested, with the exception of *syx* and *TpnC*, showed efficiency values between 95% to 105% and standard curve correlation coefficients of 0.99 (Table 1; Supplementary Fig. S1 online; Supplementary Table S1 online), meaning that the amount of PCR product approximately doubles each cycle^19^. For all genes, the melting curves showed one single peak, indicating that assays led to specific amplifications (Supplementary Fig. S1 online). Because *syx* and *TpnC* failed to show acceptable amplification efficiency (>105%), they were disregarded as reference genes, leaving six genes that were further evaluated.

### Candidate genes with relative expression stability across life stages

**Figure 1:**
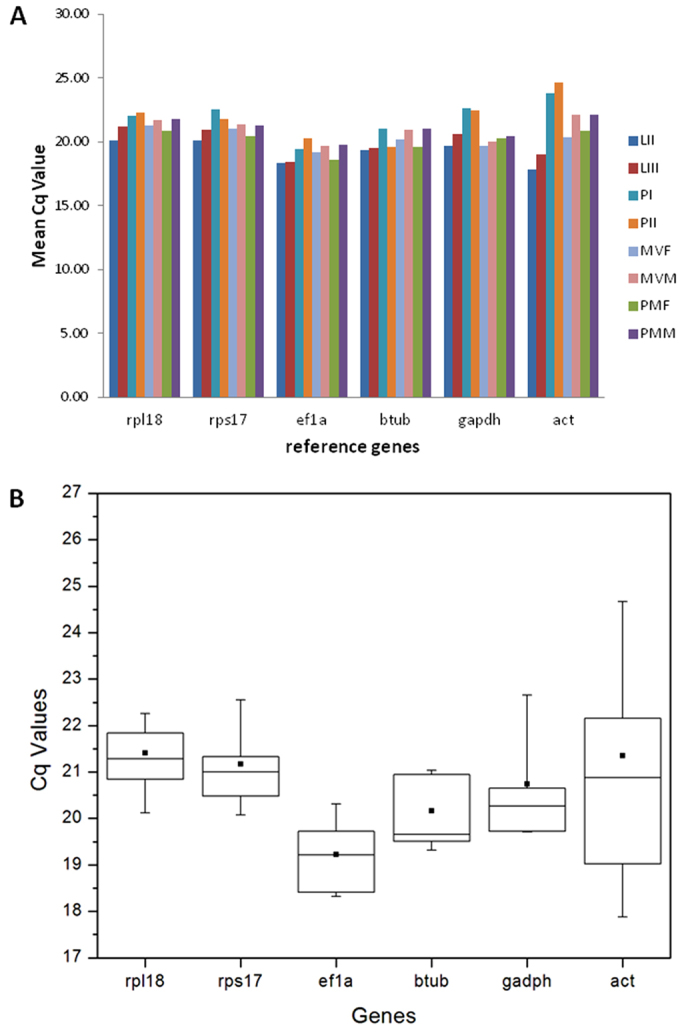
Gene expression stability of candidate reference genes. (**A**) Gene expression across different *Anastrepha obliqua* life stages. The bars show mean C_*q*_ values for each life stage from three biological replicates. (**B**) Variation in expression levels of candidate reference genes. Box-plots show medians (lines), means (dots), 95th percentile (boxes) and ranges (whiskers); (n = 24). The variation in expression level for each candidate gene was estimated across eight different life stages from *Anastrepha*, each stage with three biological replicates. The genes and their respective acronyms are: *ribosomal protein L18* (*rpl*18), *β*-*Tubulin* (*btub*), *elongation factor *1*α* (*ef*1*a*), *ribosomal protein S17* (*rps*17), *glyceraldehyde 3-phosphate dehydrogenase* (*gapdh*) and *actin* (*act*). LII - larval stage II; LIII - larval stage III; PI - pupal stage I; PII - pupal stage II; MVF - mature virgin female; MVM - mature virgin male; PMF - post-mating female; PMM - post-mating male.

The C_*q*_ (quantitative cycle) values generated from the six candidates for reference genes were used to estimate stability of gene expression across different life stages. Figure 1A,B reveals different patterns of expression and variation among candidate genes. The difference in average C_*q*_*s* among the candidate genes was only 2.18 cycles, with the lowest C_*q*_*s* values (19.23) for *ef1a* gene and the highest (21.41) for *rpl18* gene (Fig. 1A). The investigation of variation across different life stages revealed two different groups (Fig. 1B): one group consisted of *rpl18*, *rps17*, *ef1a* and *btub* with standard deviation (SD) < 1.0; and another group consisted of *gapdh* e *act* with SD > 1.0. There are several different algorithms that investigate patterns of expression to identify the best reference genes which are available for _*q*_PCR studies. In this study, by using three tools (NormFinder, BestKeeper and RefFinder) available online, we identified the most stable genes as being *rpl18*, *rps17* and *ef1a* (Table 2). It is worth noting that all three algorithms indicated that *act* exhibited the largest variation and lowest stability, and is therefore not suitable as a reference gene for the experimental conditions used in this study.

### Investigation on target genes involved in reproduction

We used the results generated from differential expression of transcripts of virgin mature and post-mating males of *Anastrepha fraterculus* transcriptome to select some differentially expressed genes. The rationale was that this difference indicates genes potentially involved in the reproductive process. Of the differentially expressed genes obtained in our in silico analysis, three OBPs were selected to represent a gene family already associated with the reproduction process^8^,^20^. Table 3 shows the differentially expressed genes selected from the *Anastrepha* transcriptome. Based on the fold-change differences, two OBP genes were chosen for being supposedly up-regulated (*Obp19d_2* and *Obp99c*) and one down-regulated (*Obp56a*) by mating.

### Expression profiles of OBPs in different life stages

Despite the important role of OBPs in the reproductive behavior of Diptera, we still lack greater knowledge about the function and expression patterns of these proteins at different developmental stages of insects. Thus, we used _*q*_PCR to determine the expression profiles of the three selected OBPs in *A. obliqua* larvae, pupae, and mature virgin and post-mating adults of both sexes. In order to generate OBP sequences from *A. obliqua*, we used *A. fraterculus* contig sequences to create primers for cloning and sequencing these genes from *A. obliqua*. This procedure generated full OBP sequences from *A. obliqua* from which we designed qPCR primers to study *A. obliqua* expression. Sequences from *A. obliqua* were very similar to OBPs from *A. fraterculus*, sharing 100% similarity for *Obp19d_2*, 97% for *Obp56a* and 93% for *Obp99c* at the amino acid level. Furthermore, the region we used to derive the qPCR primers had 100% similarity between the species, so it is likely that these primers could be used to investigate expression studies in both species, though we have only tested them for *A. obliqua*. The sensitivity of each primer set determined by standard curves with seven dilutions is shown in Table 4. These primers showed efficiency close to 100%, and standard curve correlation coefficients of 0.99 (Supplementary Fig. S2 online; Supplementary Table S2 online). The specificity of each primer set was confirmed by single melting peaks (Supplementary Fig. S2 online). For gene expression analysis, reactions were performed with three technical replicates of three biological replicates for each life stage. The relative normalized expression was calculated by 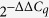 method^21^ in contrast with three validated reference genes expression (*rpl18*, *rps17* and *ef1a*). 

**Figure 2:**
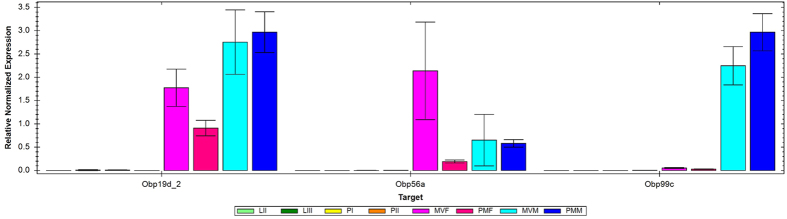
Relative normalized expression of *Obp*19*d*_2, *Obp*56*a* and *Obp*99*c* through different *Anastrepha* life stages. LII - larval stage II; LIII - larval stage III; PI - pupae stage I; PII - pupae stage II; MVF - mature virgin female; MVM - mature virgin male; PMF - post-mating female; PMM - post-mating male.

The relative transcript abundance of OBPs in larvae II, larvae III, early pupae, late pupae, mature virgin and post-mating female and mature virgin and post-mating male is exhibited in Fig. 2. All three OBP genes showed low expression during immature stages (pupae and larvae) compared to adult phases. We failed to detect significant differences in *Obp19d_2* expression between virgin and post-mating male or female adults. However, we observed a higher expression of this OBP in post-mating males when compared to post-mating females p (<0.05). As for *Obp56a*, a 2-fold decrease in abundance (unpaired t test, p < 0.01) was observed in post-mating females compared to virgin females, whereas no significant change was detected in males among different profiles. The levels of expression of this OBP in virgin and post-mating males were as low as that observed for post-mating females, but significant difference was observed only between post-mating males and females (p < 0.05). On the other hand, *Obp99c* had low expression values for mature virgin and post-mating females, comparable to the expression observed in the early stages of development. However, we observed a significantly higher expression in virgin and post-mating males in comparison to virgin and post-mating females (2-fold and 3-fold increase, respectively) (unpaired t test, p < 0.001).

## Discussion

Normalization is an essential step for the quantitative study of relative gene expression, which means that the relative expression of a target gene must be determined against a reference gene, whose expression levels should be stable and not influenced by the experimental procedure^22^. Since levels of expression in reference genes may vary depending on the species being studied, or even on environmental or developmental conditions, no reference gene should be considered universal^23^. Therefore, every _*q*_PCR experiment should begin with the identification and validation of a set of reference genes that can be reliably used according to a specific condition.

To our knowledge, this is the first study for suitable reference genes for gene expression studies in the genus *Anastrepha* across different life stages. Eight potential reference genes were analyzed and ranked according to _*q*_PCR efficiency and expression stability. From them, three (*rpl18*, *rps17* and *ef1a*) were reliable as reference genes because they showed appropriate efficiency values and constitutive expression levels across the different stages we contrasted. In fact, the efficiency and the expression stability of these three genes have already been reported for other insects. The gene *rpl18* was the most stable for the bed bug *Cimex lectularius* and the Colorado potato beetle *Leptinotarsa decemlineata*^24^,^25^. For the mosquito *Aedes aegypti*, *rps17* was the most stable gene across different ages^26^ and other experimental conditions^27^,^28^,^29^. On the other hand, *rps17* was not reliable as reference gene in three Calliphorid flies^30^. The *ef1a* gene was analyzed on *Bombus terrestris*, *Bombus lucorum*, *Bactrocera dorsalis*, *Cimex lectularius* and *Leptinotarsa decemlineata*^24^,^25^,^31^,^32^,^33^ and among these species *ef1a* was reported as a good reference gene only for *B. lucorum*. A range of mixed results for the same potential reference gene in different studies and experimental conditions reinforces the importance of search and normalization of suitable reference genes for _*q*_PCR studies. Indeed, even species from the same genus as *B. terrestris* and *B. lucorum*, have required different reference genes for _*q*_PCR studies^31^, which emphasizes the need of normalization even for closely related species.

The wide range of expression levels shown by *act* in our experiments with *A. obliqua* also highlights the importance of normalization and species-specific studies for _*q*_PCR. This gene is widely used as reference in _*q*_PCR experiments in a range of organisms, because it is assumed to be constitutively expressed. For instance, *act* is considered one of the most stable gene in dipterans such as the tephritid *B. dorsalis*^33^, and *D. melanogaster*^34^. However, our data showed that, among the potential reference genes tested, *act* had the widest range in expression values across different stages of development in *A. obliqua*, making it inappropriate as a reference gene in the conditions we considered. We should emphasize that even though *rpl18*, *rps17* and *ef1a* have been confirmed as appropriate reference genes for *A. obliqua* across the developmental stages we considered, any condition other than the ones tested here may require new validations.

An attribute that makes insects a group of great success is their sophisticated olfactory system, which is paramount to their survival and reproduction. Since we still know very little of the molecular events affecting behavior and biology of the West Indies fruit fly, information on the expression profiling of OBPs across different developmental stages and pre- and post-mating stages contributes relevant information about the role of these proteins on reproduction. Such knowledge might help towards the development of new and more environmentally friendly pest control strategies. *Obp19d_2*, *Obp56a* and *Obp99c* showed different levels of expression across development, even in immature stages (larvae and pupae), despite the very low relative transcript abundance compared to adult stages. Although several of the different expression levels here found are significant, some profiles show higher standard deviation, particularly virgin and post-mating adults in *Obp19d_2* and virgin adults in *Obp56a* (Fig. 2). Because these deviations are consistent across technical replicates, we believe that they are due to variation in expression among individuals. OBPs belong to an epistatic network of pleiotropic genes that are plastic, dynamic and modulated by several factors, such as sex, physiological state, social relationships, as well as by the environment^35^. Though we may be able to control several of these aspects in the lab, we cannot control for population variation, since we performed our study in non-inbred populations. Variation at the population level for OBP genes and their expression might be adaptive, since it might enable different response to important environmental cues. The combination of population variation, epistatic interactions, and environmental effects makes it a daunting task to investigate OBP expression, but our results still show relevant biological associations. 

In *D. melanogaster*, microarray gene expression studies showed that *Obp19d* had an adult-biased expression, as observed in our data, whereas *Obp56a* had a larval-biased expression, which differs from our results. As for *Obp99c*, high expression levels were observed in both larvae and adults of *D. melanogaster*^11^, which differs from *A. obliqua* since the expression was very low in immature stages as well as in female adults. Overall, the low levels of expression of these OBPs in immature stages of *A. obliqua* lead us to speculate that, at least in larvae, these genes seem not be involved with some basic biological needs such as nutrient sensing. Furthermore, the adult-biased expression observed for these OBP genes in *A. obliqua* may be related to the search for partners and oviposition sites.

In adults, chemically-induced changes on females due to mating, mainly related to seminal fluid proteins, have been well characterized in *D. melanogaster*^36^,^37^,^38^; as well as mating consequences in males, like immunological sexual dimorphism^39^ and stimulation of male accessory glands proteins^40^. For *A. obliqua*, we found a difference in expression levels between virgin and post-mating adults only for *Obp56a* in females, with a significant lower level of expression before mating. This difference may indicate possible regulation by mating and it could contribute to changes on female’s behavior as it happens in *D. melanogaster* females that, after sperm contact due to mating, experience a decrease in attractiveness and receptiveness to other males^37^. In fact, *Obp56a* is regulated on females by sperm contact in *D. melanogaster*^36^. In *C. capitata*, females experience a switch on certain olfactory-mediated behaviors after mating: virgin females choose the odor of male pheromone instead of volatile host plant emissions and maintain this preference even without contact with males, whereas mated females have a stronger preference for host plant odors to oviposit^41^. Thereby, changes in chemoreception genes may be expected due to mating and, although the function of *Obp56a* is not known, the decrease of its abundance in *A. obliqua* post-mating females may suggest that this protein would be more related to perception of male pheromones, or other aspects related to mating, instead of the search for oviposition sites.

Furthermore, *Obp56a* also showed sexual dimorphism in expression, since it had a higher expression on post-mating males than on females. The same was observed on mated males for *Obp19d_2* and *Obp99c*. This differential expression may be a consequence of the different gender response to the chemical environment. This sexual dimorphism is expected since males and females may have very different chemical cues. The higher expression of OBPs on post-mating males would suggest that these genes could be related to seeking partners, responding to female’s pheromone, while post-mating females gear their efforts to seeking oviposition sites, and therefore may be more responsive to volatile host plant emissions. *Obp19d* is also known as pheromone binding protein-related protein (*PBPRP2*), indicating its role with reproduction in *D. melanogaster*^42^. On the other hand, *Obp99c* was significantly more expressed on virgin and mated males suggesting that this protein may be related to functions restricted to this gender. Genes in the *Obp99* class have been associated with the smell of benzaldehyde in *D. melanogaster*^43^,^44^, which is common to several plants, especially in flowers and fruits^45^, making it a possible class of genes associated with host preference. Furthermore, benzaldehyde increased attraction of males to sex pheromone lures in *Spodoptera exigua* by over 100%^46^, making it possible that *OBP99c* is a gene involved with host and mating preference. A widespread sexual dimorphism in chemosensory genes expression, including OBPs, was documented in *D. melanogaster*, in response to different environmental, physiological and social conditions^11^. Indeed, responses to different odorants have been observed as sexually dimorphic^47^.

The results here obtained make *Obp99c* a good candidate for the application in techniques that use genetic manipulation like the Sterile Insect Technique (SIT), since its relative expression was almost exclusive to males. Likewise, the high expression of *Obp56a* on virgin females may be an important factor involved in their search for males and or mating sites. Some OBPs like *Obp19d_2*, *Obp56a* and *Obp99c*, may regulate parts of sexual behavior such as the search for hosts and sexual partners, therefore, an improvement on the knowledge about these proteins in agricultural pests like *A. obliqua* impacts not only the understanding of their biology, but may contribute to future research on the use and manipulation of OBPs for the development of new environmentally friendly pest control strategies. Furthermore, this knowledge may aid on the development of novel species-specific attractants and repellents, lures and traps for pest control programs.

## Methods

### Sampling

Flies were obtained from an *A. obliqua* population maintained in natural photoperiod at 28 °C ± 2.0 °C in the Population Genetics and Evolution laboratory, at the Federal University of São Carlos, Brazil. Different stages of development were used: 2^*nd*^ and 3^*rd*^ larval instars (LII and LIII) taken from mangoes offered to the population for oviposition, using mean standard length of each instar (3–5 mm for 2^*nd*^ instar and around 8.6 mm for 3^*rd*^ instar)^48^; early pupal stage (PI), taken around 24 hours after pupation, later pupal stage (PII), taken 10 days after pupation; sexually mature virgin males (MVM) and females (MVF), taken 10 days after adult emergence; and post-mating males (PMM) and females (PMF), taken 24 hours after mating. For each stage, three independent biological replicates were produced from pools of five different individuals.

### RNA isolation and cDNA synthesis

Total RNA was extracted using the TRIzol/chloroform protocol^49^. RNA quality was visualized in an agarose gel electrophoresis for integrity, absorbance was measured in Nanodrop (Thermo Scientific^*TM*^) and then RNA was quantified using a Qubit^®^ 2.0 Fluorometer (Invitrogen^*TM*^). The extracted RNA was treated with DNase I Amplification Grade (Invitrogen^*TM*^) prior to reverse transcription. One *μ*g of treated RNA was converted into cDNA using iScript^*TM*^ cDNA Synthesis Kit (Bio-Rad). To perform the assays, all cDNA samples were diluted to 4 ng/*μ*l. The samples were stored at −80 °C until used.

### Candidate genes and primer design

For the validation of reference genes, eight constitutive genes were chosen, most of them already used as reference for qPCR experiments in insects^24^,^31^,^32^,^33^,^50^,^51^. In order to obtain DNA sequences for *A. obliqua* for these genes, *D. melanogaster’s* sequences from the following genes were selected from GenBank database: *rpl18* (NM_139834.2), *btub* (EU980443.1), *ef1a* (GU339154.1), *rps17* (NM_079278.2), *gapdh* (GU269901.1), *syx* (NM_168905.1), *tpnC* (EZ423429.1) and *act* (L12254.1). These sequences were used to BLAST against an *A. fraterculus* transcriptome (Rezende, V.B.; Congrains, C.; Lima, A.L.; Campanini, E.B.; Nakamura, A.M.; Oliveira, J.L.; Chahad-Ehlers, S.; Sobrinho-Jr., I.; de Brito, R.A.; unpublished data) that was the only transcriptome data available at that time in our lab. We did not consider this to be much of a problem, because *A. fraterculus* and *A. obliqua* are closely related species and these genes are evolutionarily very conservative. We performed a differential expression analysis between mature virgin male and post-mating male in the *A. fraterculus* transcriptome data set using the software edgeR following default specifications^52^ and identified three differentially expressed OBP genes (*Obp19d_2*, *Obp56a* and *Obp99c*) between these profiles. These genes were chosen following the premise that the difference in expression indicates that the gene may play an important role in reproduction. We used inferred amino acid sequences for each of these OBPs to perform a BLAST search on GenBank with the best close match options to *C. capitata*, which identified the sequences *Obp19d_2* (XM_004524978.1), *Obp56a* (XM_004517843) and *Obp99c* (XM_004521129.1). We aligned these sequences to their orthologous copies obtained from the *A. fraterculus* transcriptome using MAFFT^53^ and designed primers to amplify conserved regions of each gene in *A. obliqua*. These PCR products were PEG purified and sent to a specialized company (MACROGEN INC., South Korea) to carry out the sequencing. Based on the Sanger sequences, _*q*_PCR primers were designed in regions with 100% of similarity between each *A. obliqua* sequence and *A. fraterculus* contig, using in flanking intron-exon junctions if possible, and checked for duplex formation and secondary structures with the OligoAnalyzer tool (http://www.idtdna.com/analyzer/applications/oligoanalyzer/).

### _*q*_PCR

For _*q*_PCR assays, *SsoFast*^*TM*^ Evagreen^®^ Supermix (Bio-Rad) was used with final primer concentration of 0.3 *μ*M in a total reaction of 10 *μ*l. Reactions were performed in a CFX96 TouchTM Real-Time PCR Detection Systems (Bio-Rad) using the following protocol: 95° for 30 seconds followed by 40 cycles of dissociation at 95 °C for 10 seconds and annealing and extension at 60 °C for 1 min. For melt curve analysis we used a protocol with temperatures that varied from 65 °C to 95 °C with increments of 0.5 °C for 5 seconds and continuous fluorescent measurements. The efficiency of the reactions at high and low concentrations were assessed to ensure that primer efficiency would not vary significantly due to variations in cDNA concentration among samples. Therefore, efficiency of _*q*_PCR (E) and the correlation coefficients (R^2^) were calculated for each pair of primer using standard curves of correlation between cDNA concentration and Cq values. They were generated with a serial dilution (7x) of a pool with all cDNA samples with an initial concentration of 4 ng/*μ*l, in triplicate. Moreover, to check for the uniform expression of reference genes across life stages, which is necessary for the gene stability calculation and to calculate the relative normalized expression for OBPs, assays were performed in triplicate, using each biological replicate at a concentration of 4 ng/*μ*l. No-template control (NTC) was used in all _*q*_PCR experiments to confirm that there was no contamination with exogenous material.

### Gene stability analyses of reference genes

The C_*q*_ average values calculated for the candidate reference genes in CFX Manager^*TM*^ software (Bio-Rad) were analyzed by three different descriptive statistic tools freely available online: NormFinder software (http://moma.dk/normfinder-software), BestKeeper software (http://www.gene-quantification.de/bestkeeper.html) and RefFinder web-based tool (http://www.leonxie.com/referencegene.php). NormFinder calculates not only the overall variation of the candidate normalization genes but also the variation between sample subgroups of the sample set^54^. The software BestKeeper estimates the correlation of expression levels of all candidates and the genes more stable are combined in an index. Three main indicators were used to determinate the best candidate: standard deviation, covariance and correlation analysis^55^. RefFinder ranks all candidates of reference genes based on the main algorithms currently used, including Normfinder and BestKeeper, as well as the geNorm algorithm^56^ and the Comparative ΔC_*t*_ Method^57^. Thus, RefFinder allows the choice of the best candidates based on the final ranking results of different programs^58^,^59^,^60^,^61^,^62^.

### Relative normalized expression calculation of OBPs

Like the reference genes, data analysis for OBPs was also performed using CFX Manager Software^*TM*^ (Bio-Rad). The values of relative normalized expression were calculated for each gene using the 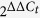 method^21^, in contrast with the three validated reference genes expression. Since experiments were performed in different plates, inter-run calibrators were used and a common threshold was set. Data were statistically compared by ANOVA and Unpaired 2-tailed t test using Prism 5.01 software (GraphPad Software, San Diego, CA, USA).

## Additional Information

**How to cite this article**: Nakamura, A.M. *et al.* Reference genes for accessing differential expression among developmental stages and analysis of differential expression of OBP genes in *Anastrepha obliqua*. *Sci. Rep.* **6**, 17480; doi: 10.1038/srep17480 (2016).

## Supplementary Material

Supplementary Information

## Figures and Tables

**Table 1:** Eight candidates for reference gene in expression studies by qPCR in *Anastrepha obliqua.*

Symbol	Gene Name	GeneBank Acession Number	Function	Ta (°C)	PCR Efficiency(%)	Correlation Coefficient	Forward primer	Reverse primer
*act*	*Actin*	L12254.1	structural constituent of cytoskeleton	59	95.3	0.994	TGACGATGAGGTTGCTGCTT	TTGTCCCATACCGACCATCA
*btub*	*β*-Tubulin	EU980443.1	structural constituent of cytoskeleton	59	101.3	0.995	CCCTCACCCAAAGTATCAGAC	TGGTCAATTTCAGAGTGCGG
*ef*1*a*	*Elongation factor*-1*α*	GU339154.1	translation elongation factor activity	59	100.0	0.999	TTGCCTTCGTACCAATCTCTG	AACCTTCCATCCCTTGAACC
*gapdh*	*Glyceraldehyde* 3 *phosphate dehydrogenase*	GU269901.1	(NAD+) (phosphorylating) activity	58	98.7	0.995	AAACTGTGGCGTGATGGAC	GACTGAGACATTGGGTGTGG
*rpL*18	*Ribosomal proteinL*18	NM139834.2	structural constituent of ribosome	58	99.5	0.996	GTGCCTAAGATGACCATTTGC	GACCTCCAGCCTTAATGATACG
*rpS*17	*Ribosomal proteinS*17	NM079278.2	structural constituent of ribosome	58	103.4	0.993	GCCGCAAAGGTCATAATTGAG	GCTACTTCTTCACAAATACGCT
*syx*	*Syntaxin*	NM168905	SNAP receptor activity	58	107.2	0.935	GAAATCTACAAGAAACTCGGTGC	CACACTTGTTTGCTCCACAG
*tpnC*	*TroponinC*	EZ423429.1	calcium ion binding	58	109.1	0.998	TCCTGAAAGAGTTAGACGAT	CTCTCCTGTCATCATTTCCAT

**Table 2:** Expression stability values of the candidate reference genes through different life stages of *A. obliqua*, according to three algorithms (ranking in parentheses).

Gene	NormFinder	BestKeeper	RefFinder
*rpl*18	0.10 (1)	0.54 (1)	1.00 (1)
*rps*17	0.14 (2)	0.55 (2)	1.68 (2)
*ef*1*a*	0.40 (3)	0.59 (3)	3.00 (3)
*btub*	0.83 (5)	0.63 (4)	4.23 (4)
*gapdh*	0.73 (4)	0.91 (5)	4.73 (5)
*act*	1.65 (6)	1.82 (6)	6.00 (6)

**Table 3:** Differentially expressed genes selected as candidates for expression studies.

Gene	Symbol	logFC^a^	logCPM^b^	Most expressed in
*Odorant-binding protein 19d*	*Obp19d_2*	−3.47735	6.501031	post-mating male
*Odorant-binding protein 56a*	*Obp56a*	4.199486	8.609884	mature virgin male
*Odorant-binding protein 99c*	*Obp99c*	−4.62665	11.25094	post-mating male

^a^logFC: log_2_ fold-change; ratio of virgin and post-mating expression values.

^b^logCPM: log_2_ counts-per-million.

**Table 4:** OBP genes studied in *Anastrepha obliqua*.

Symbol	GenBank accession number	Tm (°C)	PCR efficiency (%)	Correlation coefficient	Forward Primer	Reverse Primer
*Obp19d_2*	KP939313	60	100.5	0.999	CAAATGACAAAGCTAAGTGCCTG	CATCACCATCAGCAACTGC
*Obp56a*	KP939314	60	100.1	0.994	TGGCGACAGTTCTACTCTG	GACGATAGGCAATCCAAGC
*Obp99c*	KP939315	60	100.0	0.994	CGGATGTACCAGAGGTAAACTC	ATCTTCAGCATTATTTGGGTCC

Efficiency and correlation coefficient determined by standard curves for primers of the selected genes.
