# Supplementary material for: Reference genes for accessing differential expression among developmental stages and analysis of differential expression of OBP genes in Anastrepha obliqua
Source: Sci Rep. 2016 Jan 28;6:17480. doi: 10.1038/srep17480 (PMC4730201; doi:10.1038/srep17480)
Supplement: Supplementary Information [file srep17480-s1.pdf]

Supplementary Information

Reference genes for accessing differential expression among developmental stages and analysis of differential expression of OBP genes in *Anastrepha obliqua*

Aline Minali Nakamura[1], Samira Chahad-Ehlers[1], Andre Luís A. Lima[1], Cristiane Hayumi Taniguti[1], Iderval Sobrinho Junior[1], Felipe Rafael Torres[1], and Reinaldo Alves de Brito[1,*]

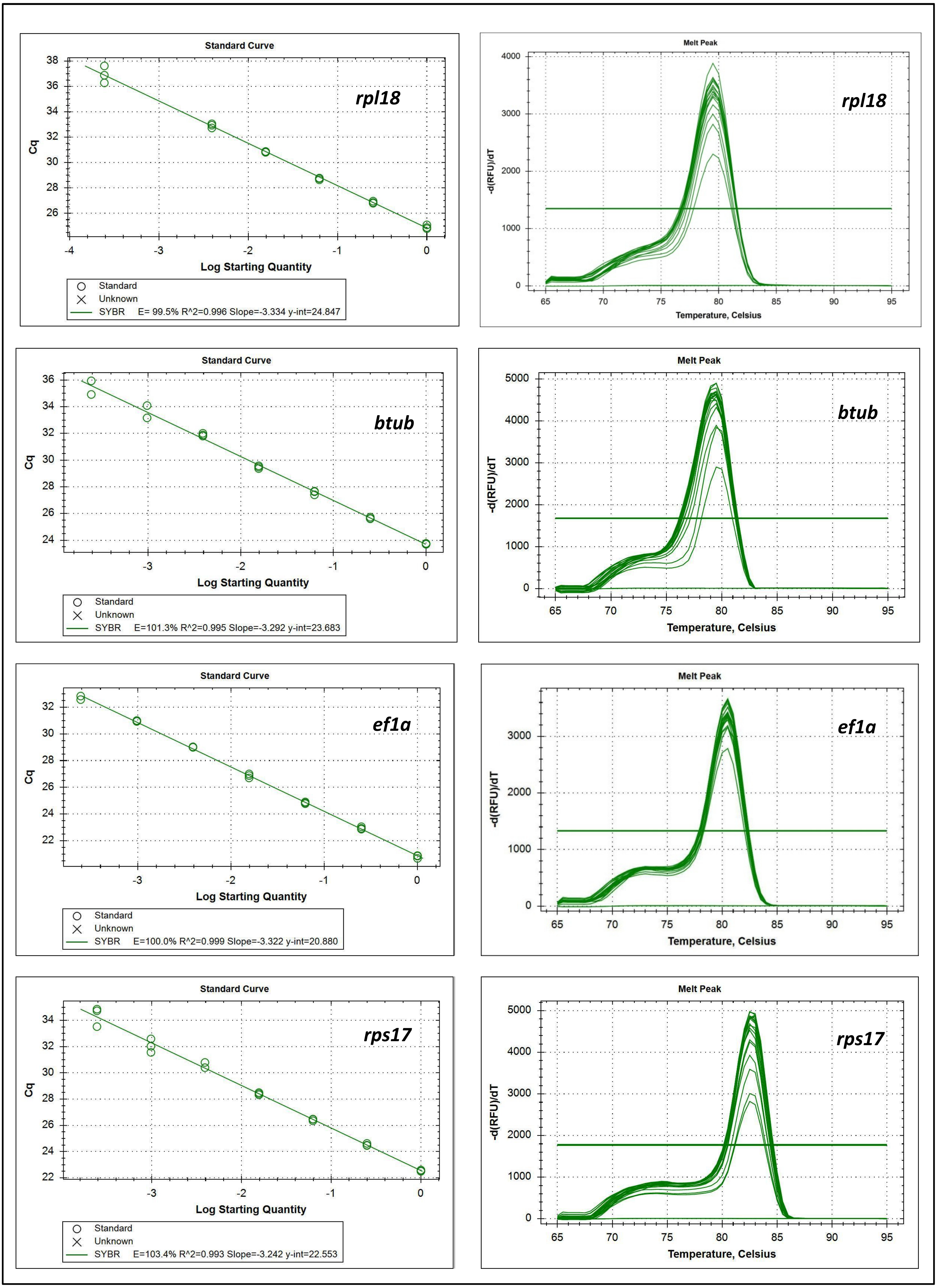
Standard Curve
rpl18
Cq
Log Starting Quantity
Standard
Unknown
SYBR E= 99.5% R^2=0.996 Slope=-3.334 y-int=24.847
Melt Peak
rpl18
-d(RFU)/dT
Temperature, Celsius
Standard Curve
btub
Cq
Log Starting Quantity
Standard
Unknown
SYBR E=101.3% R^2=0.995 Slope=-3.292 y-int=23.683
Melt Peak
btub
-d(RFU)/dT
Temperature, Celsius
Standard Curve
ef1a
Cq
Log Starting Quantity
Standard
Unknown
SYBR E=100.0% R^2=0.999 Slope=-3.322 y-int=20.880
Melt Peak
ef1a
-d(RFU)/dT
Temperature, Celsius
Standard Curve
rps17
Cq
Log Starting Quantity
Standard
Unknown
SYBR E=103.4% R^2=0.993 Slope=-3.242 y-int=22.553
Melt Peak
rps17
-d(RFU)/dT
Temperature, Celsius

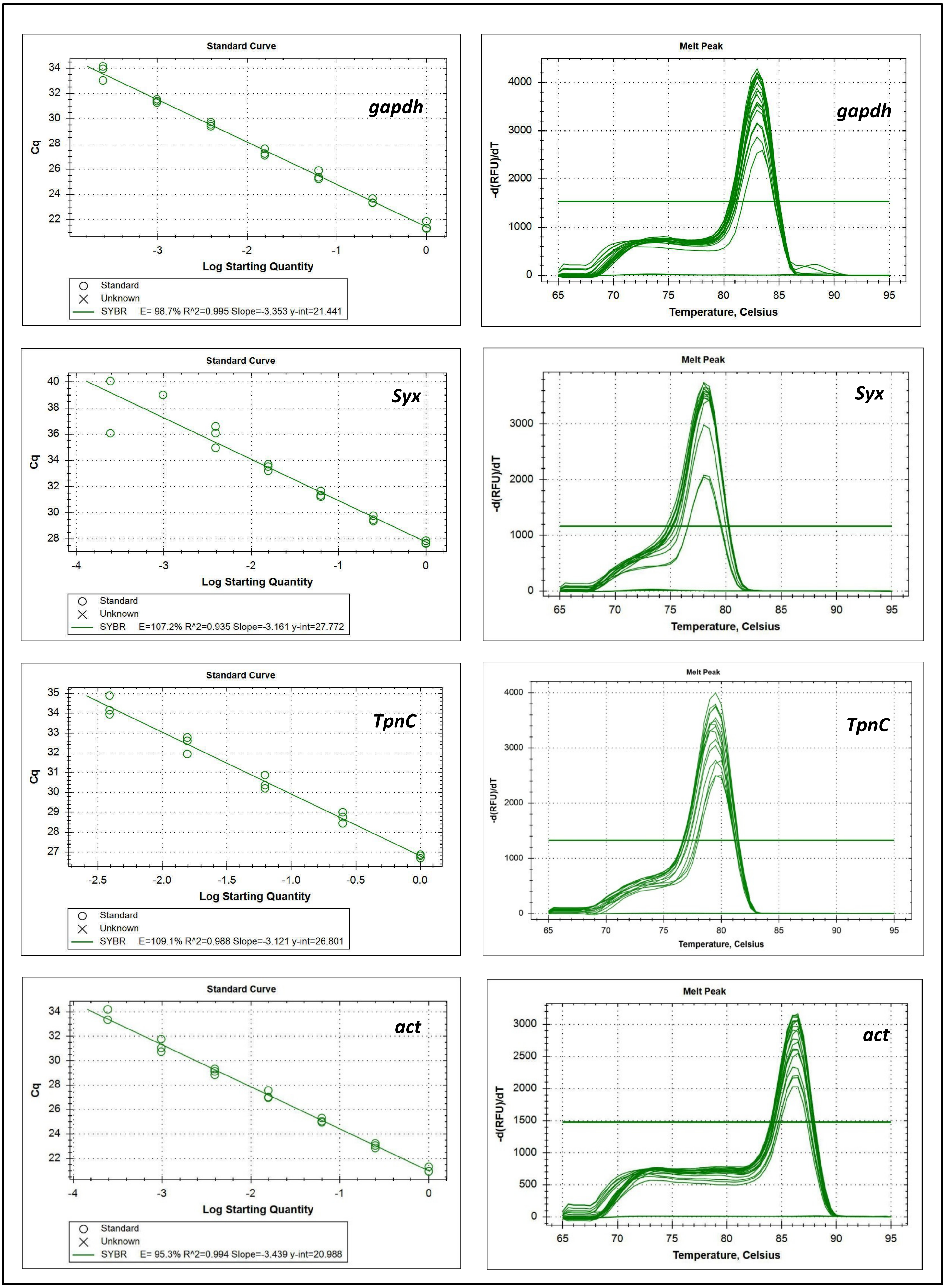
Standard Curve
gapdh
Cq
Log Starting Quantity
Standard
Unknown
SYBR E= 98.7% R^2=0.995 Slope=-3.353 y-int=21.441
Melt Peak
gapdh
-d(RFU)/dT
Temperature, Celsius
Standard Curve
Syx
Cq
Log Starting Quantity
Standard
Unknown
SYBR E=107.2% R^2=0.935 Slope=-3.161 y-int=27.772
Melt Peak
Syx
-d(RFU)/dT
Temperature, Celsius
Standard Curve
TpnC
Cq
Log Starting Quantity
Standard
Unknown
SYBR E=109.1% R^2=0.988 Slope=-3.121 y-int=26.801
Melt Peak
TpnC
-d(RFU)/dT
Temperature, Celsius
Standard Curve
act
Cq
Log Starting Quantity
Standard
Unknown
SYBR E= 95.3% R^2=0.994 Slope=-3.439 y-int=20.988
Melt Peak
act
-d(RFU)/dT
Temperature, Celsius

Supplementary Figure S1. Standard Curve and Melt Peak of all candidate reference genes. Efficiency (E) and correlation (R^2) are shown in standard curves for each genes. Melt curve graphs generated for all amplicons showing specific amplifications (only an amplified peak). The genes and their respective acronyms are: *ribosomal protein L18* (*rpl18*), *β*-Tubulin (*btub*), *elongation factor 1α* (*ef1a*), *ribosomal protein S17* (*rps17*), *glyceraldehyde-3-phosphate dehydrogenase* (*gapdh*), *syntaxin* (*Syx*), *troponin C* (*TpnC*) and *actin* (*act*).

Supplementary Table S1. The values of quantification of relative expression of the eight candidate genes in different life stages. Target = candidate genes. Sample = Life stages. Wells = replicate number for each life stage. Mean Cq = mean of the quantification cycle (Cq) for the replicate group. Cq SD = Standard deviation of the quantification cycle. The genes and their respective acronyms are: *ribosomal protein L18 (rpl18)*, *β-Tubulin (btub)*, *elongation factor 1α (ef1a)*, *ribosomal protein S17 (rps17)*, *glyceraldehyde-3-phosphate dehydrogenase (gapdh)*, *syntaxin (Syx)*, *troponin C (TpnC)* and *actin (act)*. LII – larval stage II; LIII – larval stage III; PMF - post-mating female; PMM - post-mating male; PI - pupae stage I; PII - pupae stage II; MVF - mature virgin female; MVM - mature virgin male.

Target	Sample	Wells	Mean Cq	Cq SD
act	LII	8	17.88	1.65328
act	LIII	9	19.02	1.93298
act	PI	9	23.78	1.77747
act	PII	8	24.67	0.66922
act	MVF	9	20.36	0.75382
act	MVM	9	22.11	0.76427
act	PMF	8	20.88	0.97431
act	PMM	8	22.16	0.75090
btub	LII	9	19.32	0.37517
btub	LIII	9	19.51	0.74205
btub	PI	9	21.00	1.33382
btub	PII	9	19.64	0.17510
btub	MVF	9	20.20	0.38059
btub	MVM	8	20.94	0.22500
btub	PMF	9	19.65	0.20127
btub	PMM	9	21.03	0.44043
ef1a	LII	9	18.32	1.32493
ef1a	LIII	9	18.41	0.75662
ef1a	PI	9	19.44	0.77309
ef1a	PII	9	20.31	0.58045
ef1a	MVF	9	19.21	0.75812
ef1a	MVM	8	19.72	0.75349
ef1a	PMF	9	18.62	0.72963
ef1a	PMM	9	19.79	0.53286
gapdh	LII	8	19.72	0.71701
gapdh	LIII	9	20.65	0.57882

gapdh	PI	9	22.65	2.25353
gapdh	PII	9	22.47	1.16839
gapdh	MVF	9	19.71	1.07581
gapdh	MVM	9	20.01	1.34142
gapdh	PMF	9	20.26	0.79610
gapdh	PMM	9	20.45	0.98352
rpl18	LII	9	20.12	0.96433
rpl18	LIII	9	21.24	0.89535
rpl18	PI	9	22.04	1.38822
rpl18	PII	9	22.26	1.31881
rpl18	MVF	9	21.28	1.05503
rpl18	MVM	9	21.69	1.21485
rpl18	PMF	9	20.84	1.01378
rpl18	PMM	9	21.84	1.18985
rps17	LII	9	20.07	0.70018
rps17	LIII	9	20.93	1.08797
rps17	PI	9	22.55	2.80403
rps17	PII	9	21.75	1.20290
rps17	PMF	9	21.00	0.99219
rps17	PMM	9	21.33	1.08750
rps17	PCF	9	20.48	0.91462
rps17	PCM	9	21.26	0.87976
Syx	LII	8	26.97	0.73721
Syx	LIII	9	26.13	1.56008
Syx	PI	9	26.31	2.39081
Syx	PII	9	27.12	1.50197
Syx	MVF	9	25.81	1.35919
Syx	MVM	9	26.01	1.65378
Syx	PMF	9	25.37	1.54332
Syx	PMM	9	25.91	1.14215
TpnC	LII	8	24.40	0.59521
TpnC	LIII	9	25.84	0.59481
TpnC	PI	9	28.27	2.57887
TpnC	PII	9	24.91	1.43199
TpnC	MVF	9	24.29	1.56062
TpnC	MVM	9	25.66	2.02865
TpnC	PMF	9	26.22	1.73723
TpnC	PMM	9	25.74	1.42061

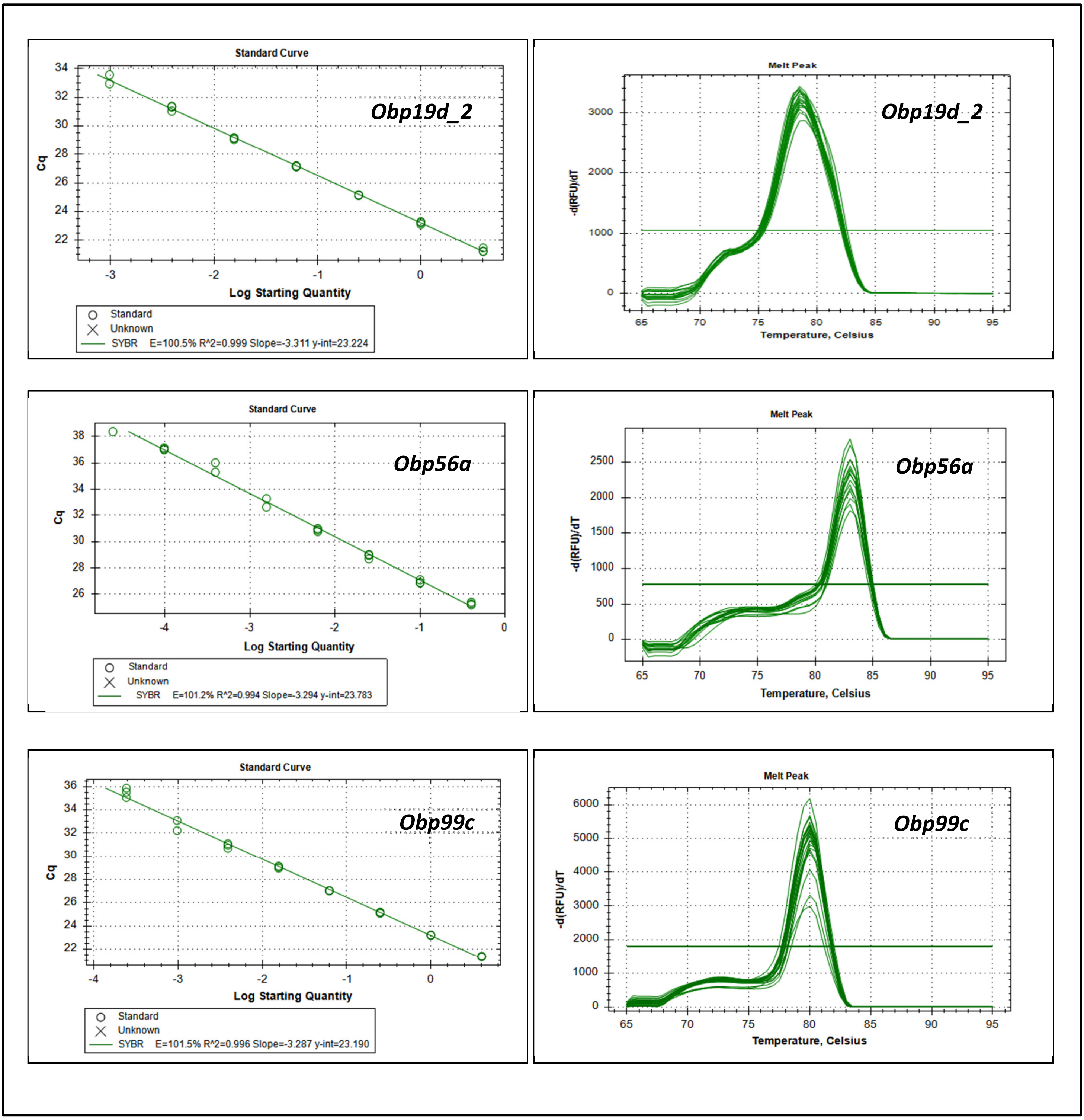

Supplementary Figure S2. Standard Curve and Melt Peak of three OBP genes (*Obp19d_2*, *Obp56a* and *Obp99c*). Efficiency (E) and correlation (R^2) are shown in standard curves for each genes. Melt curve graphs generated for all amplicons showing specific amplifications (only an amplified peak).

Supplementary Table S2. The values of quantification of relative expression of the OBP genes in different life stages. Target = candidate genes. Sample = Life stages. Wells = replicate number for each life stage. Expression = normalized gene expression (▲▲Cq). Expression SD = standard error of the mean. Mean Cq = mean of the quantification cycle (Cq) for the replicate group. Cq SD = Standard deviation of the quantification cycle. LII – larval stage II; LIII – larval stage III; PMF - post-mating female; PMM - post-mating male; PI - pupae stage I; PII - pupae stage II; MVF - mature virgin female; MVM - mature virgin male.

Target	Sample	Wells	Expression	Expression SD	Mean Cq	Cq SD
Obp19d_2	LII	8	0.00004	0.00007	34.73	2.56386
Obp19d_2	LIII	8	0.00795	0.03252	27.86	5.88018
Obp19d_2	PI	8	0.00638	0.01979	28.73	4.43363
Obp19d_2	PII	9	0.00073	0.00096	32.55	1.77706
Obp19d_2	MVF	9	1.75615	1.89400	20.37	1.45653
Obp19d_2	PMF	9	0.89122	0.94422	20.84	1.43846
Obp19d_2	MVM	9	2.68632	2.98569	20.19	1.48321
Obp19d_2	PMM	9	2.96757	2.57755	20.09	1.13750
Obp56a	LII	8	0.00038	0.00029	37.03	0.94936
Obp56a	LIII	9	0.00061	0.00056	37.15	1.22714
Obp56a	PI	9	0.00140	0.00124	36.50	1.12080
Obp56a	PII	8	0.00449	0.00314	35.52	0.78736
Obp56a	MVF	9	2.13815	3.14295	25.67	2.04885
Obp56a	PMF	9	0.19277	0.09486	28.63	0.48676
Obp56a	MVM	9	0.65172	1.65724	27.82	3.61761
Obp56a	PMM	9	0.58274	0.24222	28.02	0.28858
Obp99c	LII	8	0.00002	0.00001	33.06	0.75546
Obp99c	LIII	9	0.00004	0.00003	32.85	0.71223
Obp99c	PI	9	0.00007	0.00006	32.79	1.24074
Obp99c	PII	6	0.00177	0.00085	28.75	0.28720
Obp99c	MVF	8	0.05304	0.03699	22.89	0.84426
Obp99c	PMF	8	0.02869	0.01910	23.27	0.80937
Obp99c	MVM	9	2.26554	1.26153	17.90	0.52358
Obp99c	PMM	9	2.96757	1.21530	17.56	0.26972
